# Correction to “Implementing Pain, Agitation, Delirium, and Sleep Deprivation Protocol in Critically Ill Patients: A Pilot Study on Pharmacological Interventions”

**DOI:** 10.1111/cts.70363

**Published:** 2025-09-16

Luetrakool P, Taesotikul S, Susantitapong K, Suthisisang C, Morakul S, Sutherasan Y, Tangsujaritvijit V, Dilokpattanamongkol P. Implementing pain, agitation, delirium, and sleep deprivation protocol in critically ill patients: A pilot study on pharmacological interventions. *Clin Transl Sci*. 2024 Mar;17(3):e13739. doi: 10.1111/cts.13739. PMID: 38421247; PMCID: PMC10903435.

In Paragraph 3 of the “Study design” section, the following sentence was published with an incorrect approval number:

“This study was approved by the ethical clearance committee on human rights related to research involving human subjects, Faculty of Medicine, Ramathibodi Hospital, Mahidol University, with approval number MURA2020/150.”

This should have read:

“This study was approved by the ethical clearance committee on human rights related to research involving human subjects, Faculty of Medicine, Ramathibodi Hospital, Mahidol University, with approval number MURA2020/151.”

We apologize for this error.

